# Morbidity and mortality after liver surgery for colorectal liver metastases: a cohort study in a high-volume fast-track programme

**DOI:** 10.1186/s12893-021-01301-4

**Published:** 2021-07-14

**Authors:** Charlotte Egeland, Andreas Arendtsen Rostved, Nicolai Aagaard Schultz, Hans-Christian Pommergaard, Thomas Røjkjær Daugaard, Line Buch Thøfner, Allan Rasmussen, Jens G. Hillingsø

**Affiliations:** Department of Surgical Gastroenterology and Transplantation, Rigshospitalet, University of Copenhagen, 2100 Blegdamsvej, Denmark

**Keywords:** Colorectal liver metastases, Oncological surgery, Surgical complications, Morbidity after surgery, Lenght of stay

## Abstract

**Background:**

For colorectal liver metastases, surgery is a high-risk procedure due to perioperative morbidity. The objective was to assess severity of complications after fast-track liver surgery for colorectal liver metastases and their impact on morbidity and mortality.

**Methods:**

All patients were treated according to the same fast-track programme. Complications were graded according to the Clavien–Dindo classification for patients undergoing surgery from 2013 to 2015. Correlation between complications and length of stay was analysed by multivariate linear regression.

**Results:**

564 patient cases were included of which three patients died within 3 months (0.53%, 95% CI: 0.17–1.64%). Complications were common with Grade ≤ 2 in 167 patients (30%) and ≥ Grade 3a in 93 (16%). Patients without complications had a mean length of stay of 4.1 days, which increased with complications: 1.4 days (95% CI: 1.3–1.5) for Grade 2, 1.7 days (1.5–2.0) for Grade 3a, 2.3 days (1.7–3.0) for Grade 3b, 2.6 days (1.6–4.2) for Grade 4a, and 2.9 days (2.8–3.1) for Grade 4b. Following were associated with increased length of stay: complication severity grade, liver insufficiency, ascites, biliary, cardiopulmonary, and infectious complications.

**Conclusions:**

Complications after liver surgery for colorectal liver metastases, in a fast track setting, were associated with low mortality, and even severe complications only prolonged length of stay to a minor degree.

**Supplementary Information:**

The online version contains supplementary material available at 10.1186/s12893-021-01301-4.

## Background

The surgical approach to colorectal liver metastases (CRLM) has become increasingly aggressive, resulting in more patients becoming candidates for resection, without hampering the survival rate [1–4]. For patients with resectable disease, 5-year survival has improved significantly during the last few decades, and is currently reported as high as 50% [5–10]. Due to improved surgical techniques and downstaging neoadjuvant oncological therapies, extensive surgery for advanced metastatic disease is now an option [10–12].

Complications after liver resection are common, occurring in 4 to 48% of cases, depending on tumour type, extent of resection, and how complications are assessed [13–23]. Furthermore, complications are associated with impaired long-term survival [5, 24, 25]. To improve perioperative morbidity, hospital stay and costs implementation of enhanced recovery after surgery is a vital multimodal approach [26].

The Clavien–Dindo classification of post-surgery complications is widely accepted and validated [27, 28]. However, complications in patients undergoing resection for CRLM have yet to be described sufficiently, according to type, incidence, severity, risk factors, impact on survival, and length of hospital stay. In addition, patients resected for CRLM may differ from patients undergoing liver resection for other tumours, with respect to type of resection, chemotherapy, and comorbidity.

The aim of this study was to assess incidence, type, and severity of postoperative complications, as well as impact on length of stay and 3–6 months mortality after liver resection for CRLM in a large homogenous cohort treated in a validated, standardised enhanced recovery after surgery setting [29]. Furthermore, we aimed to identify independent risk factors.

## Methods

### Study design and patient selection

This retrospective single-centre cohort study included patients who underwent liver resection for CRLM at a tertiary referral hospital (between 1 January 2013 and 31 December 2015). All patients were evaluated for resectability at a multidisciplinary team conference to determine the best possible treatment option.

Patients were entered as cases as some patients underwent multiple liver resections.

The inclusion criteria were: Patients with CRLM undergoing liver resection or open/laparoscopic radiofrequency ablation (RFA) and age ≥ 18 years. Exclusion criteria were: combined primary colorectal resection and liver resection, other procedures in addition to liver resection (except ventral hernia repair), unresectable disease, and percutaneous RFA.

Patients were identified in the hospital surgery management system (Orbit, EVRY Healthcare Systems AB, Malmö, Sweden) with a search on all liver resection procedure codes, according to the ICD-10 codes. After the initial search, all cases were reviewed for inclusion.

This study was reported according to the “STrengthening the Reporting of OBservational studies in Epidemiology” (STROBE) statement [30].

### Fast-track programme

All patients were treated according to a previously described fast-track program based on enhanced recovery after surgery principles, with a consistent surgical approach [29] (Additional file 2: Table S1). The standard perioperative care principles in the fast-track programme are multimodal and include: standardized analgesic opioid sparing regime with epidural analgesia to POD 3, removal of nasogastric tube after surgery, removal of the abdominal drain and urine catheter at POD 1 combined with laxatives, and early mobilization. The programme is modified marginally according to laparoscopic or open surgery.

### Surgical procedure

All resections were done according to international standards favouring oncological results, with a parenchymal-sparing liver surgery philosophy. RFA was done when clinically relevant due to parenchymal sparing or comorbidity. Every surgical report was manually evaluated to ensure correct coding. We registered the number of segments resected, the number of local resections, and the number of RFAs. Furthermore, extrahepatic resections, two-stage procedures, and open or laparoscopic approaches were registered. All questionable cases were reviewed by two researchers to ensure consistency.

### Complications

Complications were graded according to the Clavien–Dindo classification [27]. Only complications Grade 2 or higher, occurring within 30 days of surgery, were registered. Grade 1 complications were not recorded, as the normal postoperative course definition is highly variable between centres according to the varying criteria for standard of care plans. Each complication was described and categorised post hoc. Two-stage procedures were treated as distinct cases with separate complications.

### Data collection

All data were collected retrospectively in 2017, using Research Electronic Data Capture (REDCap), in which all data were stored on a case level [31].

Basic data were extracted from the surgical management system and imported into REDCap. These included age, gender, and surgery time. Preoperative data collected from patient records included: the American Society of Anaesthesiologists (ASA) score, tumour type and previous liver resection. Two reviewers collected postoperative data from patient records, which involved grading of complications and length of hospital stay (from day of surgery to discharge). The method for grading complications was validated in the first 100 cases to achieve consensus. Mortality was assessed using the National Patient Register, thereby ensuring complete follow-up.

Time to discharge is defined as days from surgery to primary hospital discharge including any transfers to other hospital wards. Patients are admitted at the morning of surgery.

### Statistical analysis

The study size was determined by the number of eligible procedures in the period; pre hoc power calculation was not done. Cases with missing data were excluded from analysis. General characteristics were described as mean with standard deviation (SD) if nearly normal distributed or median with interquartile range (IQR) for continuous variables and number with percentage for categorical variables. Complications were reported as the most severe complication grade, as well as the number of complications per case. The complication categories and severity grades were described. The statistical analysis was not independent and was therefore corrected using a clustered effect.

To describe the survival rate, Kaplan Meier survival estimates were performed. The impact of complications on 6-month mortality was analysed by univariate Cox regression, with cases entered at the time of liver resection and clustered by ID.

To assess risk factors for Grade 3a or more severe complication, a univariate logistic regression was done. Significant variables in the univariate analysis were assessed in a multivariate model. Complications (independent variables) impact on the length of stay (dependent variable, natural logarithm transformed) was analysed with a multivariate linear backwards stepwise regression model. Logistic regression with a stepwise model was done to describe differences in type of complication between major and minor hepatectomy. A p-value of 0.05 or less was considered significant. Statistical analysis was done in Stata (StataCorp. 2013. *Stata Statistical Software: Release 13.* College Station, TX, USA: StataCorp LP).

### Ethics approval and consent to participate

The study was approved by the Danish Patient Safety Authority (Case Number: 3-3013-1881/1/, Reference: BELK) and by the Danish Data Protection Agency. All data in REDCap were anonymised and only the investigators had access to the patient identification key.

## Results

### Patient characteristics

In total, 957 cases were initially extracted based on procedure codes from the hospital’s surgery database. This study included 564 procedures for 462 patients, of which 373 had one, 77 had two, 11 had three, and one had four resections in the study period. The exclusion of patients are descried in Additional file 1: Figure S1. No patients were lost to follow-up at six months, with the mean follow-up at 182 days. The total follow-up was 102.492 days.

General characteristics are described in Table 1. Male gender was prominent (63%), and mean age at surgery was 67 years (SD 10). Local resection alone or combined with RFA was done in 274 (48%) cases. RFA was the only treatment in 34 (6%) of cases.

**Table 1:** General characteristics

	n = 564
Age at surgery, mean (SD)	67 (10)
Male, n (%)	353 (63%)
ASA score, missing 16	
1	49 (9%)
2	358 (65%)
3	140 (26%)
4	1 (0%)
Liver resection number	
1	406 (72%)
2	121 (21%)
3	26 (5%)
4	7 (1%)
5	4 (1%)
Surgery duration minutes, mean (SD)	158 (66)
Two-stage, n (%)	50 (9%)
Laparoscopic, n (%)	35 (6%)
Right hepatectomy, n (%)	81 (14%)
Left hepatectomy, n (%)	27 (5%)
S5 and S8 hepatectomy, n (%)	5 (1%)
S6 and S7 hepatectomy, n (%)	18 (3%)
S2 and S3 hepatectomy, n (%)	25 (4%)
One segment, n (%)	61 (11%)
Other two segments, n (%)	12 (2%)
Other three segments or more, n (%)	26 (5%)
Local resection only, n (%)	274 (48%)
One local resection, n (%)	142 (52%)
Two local resections, n (%)	70 (26%)
Three local resections, n (%)	31 (11%)
Four or more local resection, n (%)	31 (11%)
Only RFA, n (%)	34 (6%)
Extrahepatic resection, n (%)	39 (7%)
Length of stay, median (IQR), missing 25	4 (3–6)
Preoperative bilirubin, median IQR, missing 169	7 (5–10)
Preoperative ALAT, median IQR, missing 170	23 (18–34)

General characteristics of the population. Length of stay is defined as day of surgery to discharge. *N* number, *RFA* radio-frequency ablation, *ASA* American Society of Anaesthesiologists score, *IQR* interquartile range, *S* segment, *ALAT* alanine transaminase

### Complications

**Fig. 1:**
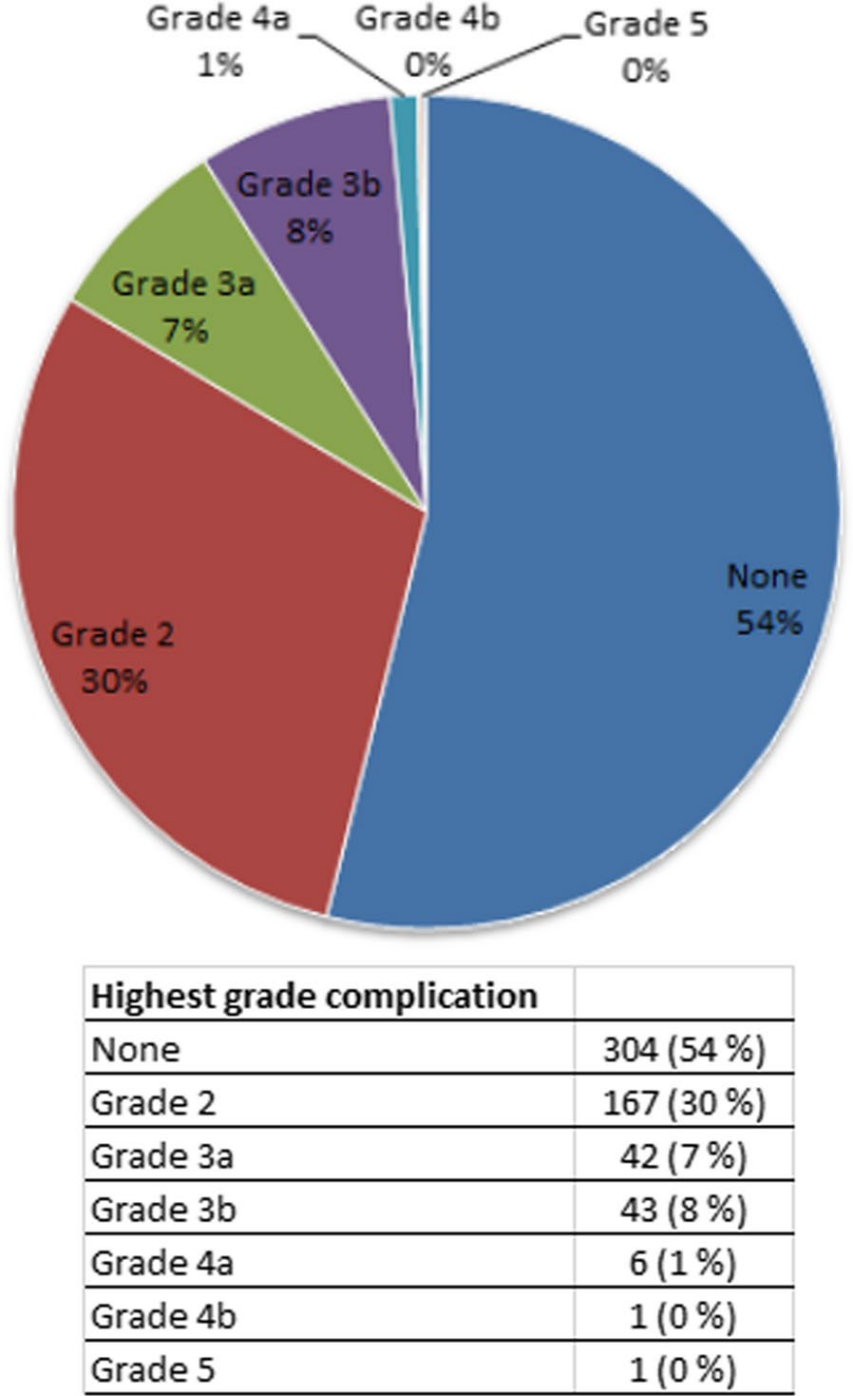
Distribution of highest grade complication. Highest grade complication after liver surgery for colorectal liver metastases according to Clavien–Dindo classification

Overall morbidity (≥ Grade 2) within 30 days of surgery occurred in 260 cases (46%). Figure 1 describes complication grades further, most notably 93 cases (16%) had a complication of Grade 3 or higher. More than half, 304 (54%) cases, had no ≥ Grade 2 complication, 157 (28%) had one, 58 (10%) had two, 28 (5%) had three, and 17 (3%) had four or more. All complications were categorised in Table 2. The merely surgical complications occurred as wound complications in 58 cases (10%), biliary complications in 25 (4%), other surgical issues in 8 (1%), and bleeding in 7 (1%), with a total of 88 (16%) cases. Wound dehiscence occurred in 18 (3%) cases. A complete list of complications, and their categorisation, is shown in Additional file 2: Table S1.

**Table 2:** Description of complications

	Total	Grade 2	Grade 3a	Grade 3b	Grade 4a	Grade 4b	Grade 5
Infection, n (%)	95 (17%)	92 (97%)	–	1 (1%)	2 (2%)	–	–
Biliary, n (%)	25 (4%)	2 (8%)	19 (76%)	4 (16%)	–	–	–
Post-operative bleeding, n (%)	7 (1%)	4 (57%)	2 (29%)	1 (14%)	–	–	–
Cardio-pulmonary, n (%)	29 (5%)	14 (48%)	11 (38%)	-	4 (14%)	–	–
Liver insufficiency, n (%)	59 (10%)	57 (97%)	1 (2%)	-	-	1 (2%)	–
Anemia, n (%)	44 (8%)	44 (100%)	–	–	–	–	–
Gastrointestinal, n (%)	48 (9%)	48 (100%)	–	–	–	–	–
Wound, n (%)	58 (10%)	6 (10%)	16 (28%)	36 (62%)	–	–	–
Ascites, n (%)	11 (2%)	8 (73%)	2 (18%)	1 (9%)	–	–	
Other surgical, n (%)	8 (1%)	–	1 (13%)	6 (75%)	–	–	1 (13%)
Other medical, n (%)	34 (6%)	30 (88%)	4 (12%)	–	–	–	–

Description of complications by total and each grade in numbers and percent. N, number

Patients undergoing open RFA had following complications: eight patients had a grade 2 complication (24%),  two patients had a grade 3a complication (6%), and five patients had a grade 3b complication (15%). Out of the seven patients who had a grade 3 or more complication, all had a wound complication.

### Risk factors for severe complications

Complications of ≥ Grade 3a were considered severe, and risk factors associated with these are listed in Table 3. Type of surgery was analysed in seven categories, only segmentectomy of ≥ 3 segments (134, 24%) cases was associated with severe complications and was comprised to major resection in further analyses.

**Table 3:** Risk factors for severe complications

	Univariate		Multivariate	
	OR for ≥ Grade 3a (95% CI)	p-value	OR for ≥ Grade 3a (95% CI)	p-value
Age at surgery per 10 year	1.1 (0.9–1.4)	0.161		
Male	2.5 (1.5–4.2)	**0.001**	2.3 (1.4–4.0)	**0.002**
ASA				
1	Ref	Ref		
2	1.1 (0.5–2.6)	0.808		
3 or more	1.5 (0.6–3.7)	0.394		
Liver surgery number				
One, n (%)	Ref	Ref		
Two, n (%)	1.1 (0.6–1.9)	0.790		
Three or more, n (%)	2.0 (0.9–4.3)	0.066		
Surgery duration per 30 min	1.2 (1.1–1.4)	** < 0.001**	1.1 (1.0–1.3)	**0.013**
Laparoscopic	NA	NA		
Number of local resections				
One	Ref			
Two	0.4 (0.1–1.1)	0.079		
Three	0.4 (0.1–1.9)	0.260		
Four or more	1.3 (0.5–3.4)	0.541		
Type of surgery				
Local resection only	Ref	Ref		
4 or more segments	3.4 (1.9–6.0)	** < 0.001**		
3 segments	3.8 (1.7–8.7)	**0.001**		
2 segments	1.1 (0.5–2.7)	0.800		
1 segment	1.3 (0.6–3.1)	0.540		
RFA only	2.2 (0.9–5.1)	0.080		
Local resection 4 or more	1.8 (0.7–4.5)	0.190		
Major resection (≥ 3 segments)	2.9 (1.8–4.6)	** < 0.001**	2.2 (1.3–3.7)	**0.003**
Extrahepatic resection	0.6 (0.2–1.6)	0.286		
Preoperative bilirubin, logarithmic transformed	1.4 (0.9–2.4)	0.2		
Preoperative ALAT, logarithmic transformed	1.1 (0.7–1.7)	0.8		

To analyse risk factors for severe complications (≥ Grade 3a) univariate and multivariate logistic regression models were done. Major resection (≥ 3 segments) were comprised from the results of the univariate model, thus were other types of surgery not included in the multivariate model. NA (non-applicable) is used as there were no severe complications. *OR* odds ratio, *N* number, *RFA* radio-frequency ablation, *ASA* American Society of Anaesthesiologists score, *ALAT* alanine transaminase

Severe complication risk factors in the multivariate model were male gender (OR 2.4, 95% CI: 1.4–4.0,  p = 0.002), surgery duration (OR 1.1 per 30 min, 95% CI: 1.01–1.2, p = 0.04), and major resection (OR 2.4, 95% CI: 1.4–4.0,  p = 0.001). Three or more liver resections was nonsignificant (OR 1.8, 95% CI: 0.8–4.0, p = 0.1), neither was comorbidities evaluated by the ASA score (reference ASA 1; ASA 2 OR 1.1, 95% CI: 0.5–2.6; ASA 3 or more OR 1.5, 95% CI: 0.6–3.7), preoperative bilirubin (OR 1.4, 95% CI: 0.9–2.4), or ALAT (OR 1.0, 95% CI: 0.7–1.7). More men had a higher ASA score (OR 1.5 by ordinal logistic regression, 95% CI: 1.04–2.2).

### Impact on length of hospital stay

Patients without any complications had a mean length of stay of 4.1 days, which increased with complications: 1.4 days (95% CI: 1.3–1.5) for Grade 2, 1.7 days (95% CI:1.5–2.0) for Grade 3a, 2.3 days (95% CI: 1.7–3.0) for Grade 3b, 2.6 days (95% CI: 1.6–4.2) for Grade 4a, and 2.9 days (95% CI: 2.8–3.1) for Grade 4b. Only 20% of patients had a length of ≥ 6 days. Complications were associated with increased length of stay (Table 4). Following were associated with increased length of stay in the multivariate model: highest complication grade (OR 1.1, 95% CI: 1.1–1.1), infections (OR 1.2, 95% CI: 1.1–1.4), biliary (OR 1.9, 95% CI: 1.4–2.6) and cardiopulmonary complications (OR 1.3, 95% CI: 1.1–1.7), liver insufficiency (OR 1.3, 95% CI: 1.1–1.5), and ascites (OR 1.9, 95% CI: 1.5–2.3). Notably, postoperative bleeding was not significantly associated with length of stay, and occurred in seven cases (1.6%), of which one was Grade 3b.

**Table 4:** Correlation between complications and length of stay a multivariate stepwise model

Complication	Coefficient	Lower 95% CI	Upper 95% CI	p-value
Highest complication per increase	1.1	1.1	1.1	< 0.001
Type of complication				
Infection	1.2	1.1	1.4	0.005
Biliary	1.9	1.4	2.6	< 0.001
Cardio-pulmonary	1.3	1.1	1.7	0.016
Liver insufficiency	1.3	1.1	1.5	< 0.001
Anemia	1.1	1.0	1.3	0.064
Ascites	1.9	1.5	2.3	< 0.001

Analysis of the correlation between complications and length of stay (logarithmic transformed) in a multivariate stepwise (removal if p ≥ 0.1) linear regression. Following was removed from the model: Total complications (p = 0.9), bleeding (p = 0.6), other surgical (p = 0.5), wound (p = 0.6), other medical (p = 0.3), anemia (p = 0.1) and gastrointestinal complications (p = 0.1). Length of stay was defined as time from surgery to discharge. *OR* odds ratio

Patients with Grade 4 complications (n = 7) had a median length of stay of eight days (IQR 7–21). Three patients had postoperative cardiac failure with a prolonged stay in the postoperative anaesthesia care unit of 24–48 hours and received vasopressor and inotropica. They were discharged to their home after 5, 7 and 8 days, respectively. One patient had < 1 min cardiac arrest and was discharged after  seven days. One had pneumonia after aspiration and was admitted after 21 days. One had septicaemia from an intraabdominal collection, discharged after 22 days. One patient had mild liver and kidney insufficiency without need for renal replacement therapy and shortly respiratory failure discharged after 11 days, but died at day 26 after surgery.

### Predictor of short-term survival

The study includes a total of 564 cases. Three patients died within three months (mortality rate 0.5%, 95% CI: 0.2–1.6%; survival 99.5 (98.4–99.8%)) and ten patients died within six months (mortality rate 1.6%, 95% CI: 0.8–3.0%; survival 98.4 (97–99.2%)). Two deaths may be attributed to surgery: one was caused by thrombosis of the superior mesenteric artery (two days after surgery) and one by biliary leak (120 days after surgery). One died from cardiac decompensation (day 26). Three patients died from chemotherapy induced infections and multi-organ failure (days 53, 114, and 177). One patient died of sudden cardiac arrest at home (day 115). Cause of death was unknown in the last three cases (days 155, 165, and 183).

Complication Grade 3a or higher were not significantly associated with 6-month mortality (hazard ratio (HR) 3.5, 95% CI: 0.9–13.3, p = 0.07), and neither was the number of complications (HR 1.3, 95% CI: 0.95–1., p = 0.1).

### Difference in complications between major and minor liver surgery

Cases that required major hepatectomy had significantly more severe complications (OR 1.8, 95% CI: 1.4–2.2) (Additional file 2: Table S3). Patients undergoing major hepatectomy had a higher incidence of liver insufficiency (OR 10.4, 95% CI: 5.0–21.9) and ascites (OR 14, 95% CI: 1.4–137.9), but the incidence of other surgical complications  such as (OR 0.1, 95% CI: 0.01–1.0) wound complications (OR 0.2, 95% CI: 0.1–0.5) and postoperative bleeding (OR 0.3, 95% CI: 0.1–1.0) were lower.

## Discussion

In this study of fast-track colorectal liver metastasis surgery in a high-volume centre, the three months mortality rate was only 0.5%, and ≥ Grade 3a complications occurred after 16% of the procedures. Overall complications (≥ Grade 2) occurred after 46% of the procedures. Infections (17% of cases) followed by liver insufficiency (10%) and wound complications (10%) were the most common. Biliary and bleeding complications were rare but occurred in respectively 4% and 1% of cases. Complications did not increase the risk of dying within three or  six months after surgery in this study, but only three patients died within three months and ten at six  months which makes statistical analysis difficult.

The mortality rate in our study was low (0.5%, 95% CI: 0.2–1.6%). In other studies of liver resection for CRLM mortality was rare, but the rate varied considerably between centres from 1.6 to 7% [5, 32–34]. However, the complication rate in our study was not lower with overall complication rate of 46% (≥ Grade 2) and severe complications in 16%, compared with the literature with overall complication rate of 20 to 42% and severe complications in 10 to 20% of cases, but not all cases were CRLM [5, 32, 35, 36]. Improving treatment outcomes after liver resection for CRLM require benchmarking between centres, despite different thresholds for interventions when complications occur. Furthermore, chemotherapy and baseline liver function may differ between centres and bias the outcome comparison. Previous publications on fast-track liver resection from our institution showed similar complication and mortality rates, as reported in this study [29, 37].

We found that male gender, duration of surgery, and major hepatectomy were independently associated with increased risk of severe complications. None of these factors are modifiable and it is well known that large resections, longer the operation and male gender increase the risk of complications [13, 36]. Men had a higher comorbidity with a higher ASA score, but the higher risk of complications may also partly represent the fact that surgery is technically more difficult in men due to size.

The length of stay in this study was 4.1 days and 20% of patients had a length of ≥ 6 days. In our philosophy the best way to recover from surgery is at home [26]. The enhanced recovery after surgery approach (fast-track) has been successful in decreasing length of stay [29, 37]. The primary care in Denmark is publicly financed and easily accessible and patients can be discharged to selfcare at home, home care nursing which can administer parenteral medicine, or to a rehabilitation facility. After discharge patients are contacted per phone by a surgical nurse the day after discharge and 21 days after surgery. The hospital stay is merely to recover patients to the extent that home care is safe. However, comparison of hospital stay between different countries is difficult as the healthcare systems may support early discharge very different.

Not surprisingly, severity of the complication led to an increased length of stay. The surgical complications being biliary, ascites and liver insufficiency were also associated with an increased length of stay, as were the medical cardiopulmonary and infectious complications. We established the type of complications after liver resection for CRLM, which will be used to improve the fast-track strategy by improving optimization prior to surgery and postoperative care. The interventions will be discussed in a multidisciplinary team and evaluated in new studies.

The Clavien–Dindo classification is a concise and validated method, which makes it an excellent registry tool, as lack of quality may be difficult to assess if not collected in a standardised manner, based on the intervention. However, focus on intervention, rather than type of complication may limit the clinical impact, but has proven easier to register retrospectively rather than surgeon reported complications diagnoses. To improve treatment, it is vital to have detailed knowledge of each complication.

Recent studies have shown that laparoscopic liver surgery may have advantages compared to open surgery by lowering complication rates and length of stay [38]. However, in our study the length of stay was four days with the majority of patients undergoing open surgery which is similar to large cohorts with patients  undergoing laparoscopic surgery [39]. The results found in this study may illustrate that the benefit from minimal invasive surgery is not being fully utilized in centres where laparoscopic surgery is predominant. Our median length of stay in highly selected laparoscopic cases are  two days.

The study was limited by its retrospective nature. The correlation between complications and mortality could not be evaluated sufficiently, as very few cases died. Readmission rates were not available, as patients may have been hospitalized outside our region, but our overall readmission rate was between 15% and 19% during this period (local quality project, not published). Compared to other large studies, this study has a lower rate of major hepatectomies, but was also more up to date with a parenchymal sparing strategy. Grade 1 complications were not included in the study which may lead to an underrepresentation of cases with a deviating postoperative course, but in the light of the present data the influence of grade 1 complication is considered negligible also because a very extensive plan of care might hide these complications. Many potential risk factors for complications could not be investigated in our study setting, most importantly data on chemotherapy were not reported.

This material includes one of the largest cohorts on resection of colorectal liver metastases, from a modern high-volume centre for three years, using standardised surgical techniques and a fast-track approach as the standard of care. Other large studies on liver resection for CRLM were conducted over longer periods up to almost 20 years, increasing the risk of bias [5, 32–34]. We had very little missing data due to electronic medical records.

## Conclusion

In conclusion, fast-track liver surgery with the majority done as open cases, carries a low mortality, but also a severe morbidity rate, that affects the length of stay although less than anticipated by their severity. However, complications irrespective of their classification (≥ Grade 2) increase the length of stay. Fast track protocols and high volume may reduce mortality considerably, which warrants further investigation of the consequent use of fast track also in minimal invasive surgery.

## Supplementary Information

**Additional file 1.** Inclusion flowchart in the study. HCC, hepatocellular carcinoma. N, number.

**Additional file 2: Table S1.** Fast-track liver resection standard perioperative care principles. **Table S2.** All complications described. **Table S3.** Risk of complications if comparing major (≥ 3 segments, n = 134) with minor surgery (n = 430).

## Data Availability

The datasets used and/or analysed during the current study are available from the corresponding author on reasonable request according to the Danish law on Data Protection of healthcare data.

## Abbreviations

ALAT: Alanine transaminase
ASA: American Society of Anaesthesiologists score
CRLM: Colorectal liver metastases
CI: Confidence interval
IQR: Interquartile range
RFA: Radiofrequency ablation
REDCap: Research electronic data capture
OR: Odds ratio
SD: Standard deviation
STROBE: STrengthening the Reporting of OBservational studies in Epidemiology
