# Supplementary figures and images for: Morbidity and mortality after liver surgery for colorectal liver metastases: a cohort study in a high-volume fast-track programme
Source: BMC Surg. 2021 Jul 14;21:312. doi: 10.1186/s12893-021-01301-4 (PMC8278677; doi:10.1186/s12893-021-01301-4)

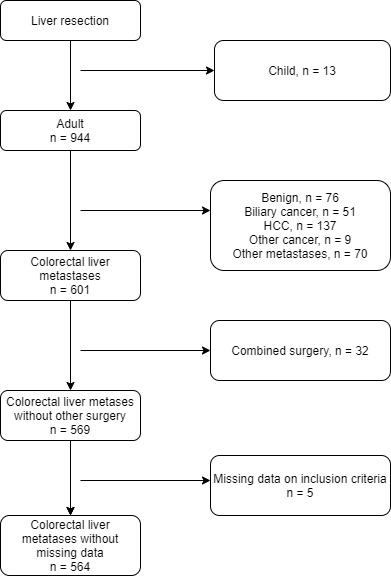

Supplement: Supplementary file 1 — Additional file 1. Inclusion flowchart in the study. HCC, hepatocellular carcinoma. N, number. [file 12893_2021_1301_MOESM1_ESM.jpg]
