# Supplementary material for: Morbidity and mortality after liver surgery for colorectal liver metastases: a cohort study in a high-volume fast-track programme
Source: BMC Surg. 2021 Jul 14;21:312. doi: 10.1186/s12893-021-01301-4 (PMC8278677; doi:10.1186/s12893-021-01301-4)
Supplement: Supplementary file 2 — Additional file 2: Table S1. Fast-track liver resection standard perioperative care principles. Table S2. All complications described. Table S3. Risk of complications if comparing major (≥ 3 segments, n = 134) with minor surgery (n = 430). [file 12893_2021_1301_MOESM2_ESM.docx]

# Additional Table S1: Fast-track liver resection standard perioperative care principles

|  | **Laparoscopic resection** | **Minor open resection** | **Major open resection** |
| --- | --- | --- | --- |
| Epidural analgesia | ÷ | POD 0-3 morning | POD 0-3 morning |
| i.v. methylprednisolone before start of surgery | + | + | + |
| Analgesics: Gabapentin + Celecoxib + Paracetamol(Gabapentin reduced in patients +65 years) | + (local analgesia in incisions) | + | + Paracetamol: individual assessment |
| Nasogastric tube removed immediately after surgery | + | + | + |
| Abdominal drain | ÷ | Optional (out POD 1) | Optional (out POD 1) |
| Sent to ward | POD 0 | POD 0 | Optional: stay at perioperative department POD 0 |
| Removal of urinary catheter | POD 0 | POD 1 (morning) | POD 1 |
| Peripheral i.v. catheter | Closed POD 0 | Closed POD 0 | Closed POD 0 |
|  | Removed POD 1 | Removed POD 1 | Removed POD 1 |
| Routine blood tests | POD 1 | POD 1 | POD 1-3 |
| Laxatives: chewing gum × 3 daily + Laxoberal drops × 1 | POD 0 | POD 0 | POD 0 |
| Mobilization | POD 0: out of bed > 2 h | POD 0: out of bed 2 h | POD 0: out of bed 2 h |
|  | POD 1: out of bed > 8 h + walking exercise × 3 | POD 1: out of bed ≥ 8 h + walking exercise × 3 | POD 1: out of bed ≥ 8 h  + walking exercise × 3 |
| Discharge | Aim at discharge POD 1-2 | Aim at discharge POD 3 | Aim at discharge POD 4 |
| On demand ordination | Ondansetron (intravenous), morphine (peroral and intravenous), furosemide (intravenous), intravenous fluids. | | |

Description of perioperative standard of care for fast-track liver resection from Schultz NA, Larsen PN, Klarskov B, et al. Second Generation of a Fast-track Liver Resection Programme (28). World J Surg. 2018;42(6):1860-1866.. POD, postoperative day

# Additional Table S2: All complications described

| **Infectious complications** |
| --- |
| Pneumonia |
| Candidiasis |
| Septicemia |
| Gastroenteritis |
| Unknown |
| Urinary tract infection |
| Herpes simplex |
| Bacteremia |
|  |
| **Biliary complications** |
| Biliary leak |
| Bile duct stones |
| Biliary absces |
| Fistula to small intestine |
|  |
| **Bleeding** |
| Liver bleeding |
| Hematoma |
| Upper gastrointestinal bleeding |
| Bleeding from drain site |
| Non-specified bleeding |
|  |
| **Cardiopulmonary complication** |
| Atrial fibrillation |
| Circulatory failure |
| Pleuritis |
| Pneumothorax |
| Hypovolemia |
| Vasovagal syncope |
| Cardiac arrest |
| Pleura effusion |
| Bundle branch block |
|  |
| **Liver insufficiency** |
| Low coagulation factor II, VII, X |
| Hypoalbuminemia |
| Hyperammonemia |
| Encephalopathy |
|  |
| **Anemia** |
|  |
| **Gastrointestinal** |
| Obstipation |
| Delayed gastric emptying |
| Total parenteral nutrition |
| Non-infectious diarrhea |
| Dyspepsia |
|  |
| **Wound** |
| Wound infection |
| Wound dehiscence |
| Fascia dehiscence |
| Hematoma |
| Bleeding |
| Seroma |
| Delayed wound healing |
| Pain during suture removal |
|  |
| **Other surgical complication** |
| A. mesenterica superior thrombosis |
| Ileus |
| Hernia |
| Iatrogenic diaphragmatic injury |
|  |
| **Ascites** |
| Drainage |
| Albumin |
| Spironolactone |
|  |
| **Other medical complications** |
| Hyperkalemia |
| Hypokalemia |
| Delirium |
| Impaired bladder emptying |
| Skin itching |
| Shoulder pain |
| Hallucination |
| Allergy |
| Spinal polysynaptic reflexes |
| Phlebitis |
| Pain |
| Hyperzincemia |
| Hyperglycemia |
| Hypoglycemia |
| Opoid poisoning |
| Hypoalbuminemia |

Description and grouping of all complications observed in the population.

# Additional Table S3: Risk of complications if comparing major (≥3 segments, n = 134) with minor surgery (n = 430)

| Complication | Odds ratio | Lower 95% CI | Upper 95% CI | P |
| --- | --- | --- | --- | --- |
| **Highest complication per increase** | 1.8 | 1.4 | 2.2 | <0.001 |
| **Type of complication** |  |  |  |  |
| Post-operative bleeding | 0.3 | 0.1 | 1.0 | 0.045 |
| Cardio-pulmonary | 0.5 | 0.2 | 1.1 | 0.08 |
| Liver insufficiency | 10.4 | 5.0 | 21.9 | <0.001 |
| Wound | 0.2 | 0.1 | 0.5 | <0.001 |
| Ascites | 14.0 | 1.4 | 137.9 | 0.02 |
| Other surgical | 0.1 | 0.01 | 1.0 | 0.05 |

Analysis of major vs minor surgery for differences in complications using multivariate stepwise logistics (removal if P>0.1) regression were done. Following was removed from the mode: Total number of complications (P = 0.9), other medical (P = 0.5), infection (P = 1.0), biliary (P = 0.2), anemia (P = 0.7) and gastrointestinal complication (P = 0.6). OR, odds ratio. CI, confidence interval. N, number.
